# Phytochemical Profiling of Leaf, Stem, and Tuber Parts of *Solena amplexicaulis* (Lam.) Gandhi Using GC-MS

**DOI:** 10.1155/2014/567409

**Published:** 2014-07-14

**Authors:** Karthika Krishnamoorthy, Paulsamy Subramaniam

**Affiliations:** PG and Research Department of Botany, Kongunadu Arts and Science College, Coimbatore, Tamil Nadu 641029, India

## Abstract

*Objective*. To explore the possible bioactive compounds in the methanolic extracts of leaf, stem, and tuber parts of the medicinal climber, *Solena amplexicaulis*, using GC-MS. *Methods*. GC-MS analysis of the plant extracts were performed by using GC-MS-5975C [Agilent] and mass spectra of the compounds found in the extract was matched with the data in the library of National Institute of Standards and Technology (NIST). *Results*. Thirty-five compounds were determined to be present in the parts studied. The active principles with their retention time, molecular formula, molecular weight, peak area, structure, category of the compounds, and activities were predicted. The most prevailing compounds were phytol (38.24%) in leaf, 4-(4-ethoxyphenyl) but-3-en-2-one (56.90%) in stem, and 9,17-octadecadienal, (Z)- (21.77%) in tuber. *Conclusion*. This study revealed that the species *S. amplexicaulis* is a good source of many bioactive compounds like terpenes, triazines, esters, alkanes, alcohols, hydrocarbons, aldehydes, amides, and so forth. That justifies the traditional usage of this species.

## 1. Introduction

Herbal plants are valuable gift of nature for mankind and they are the source of a variety of phytochemicals which are utilized for human and animal diets also. It is capable of synthesizing an overwhelming variety of low molecular weight organic compounds called secondary metabolites, usually with unique and complex structures. The medicinal actions of plants unique to particular plant species or groups are consistent with the concept that the combination of secondary products in a particular plant is taxonomically distinct [1]. It states that around 85–90% of the world's population consumes traditional herbal medicines [2]. In recent decades, studies on phytochemical constituents of medicinal plant and its pharmacological activities have received wide attention [3–6]. WHO has emphasized the need to ensure the quality of medicinal plant products using modern techniques with the application of suitable standards. Many modern methods are adapted for identification and quantification of active principle compounds in plant materials. Of them, gas chromatography-mass spectrometry (GC-MS) has become firmly established as a key technological platform for secondary metabolite profiling in both plant and nonplant species [7, 8].

The plant species* Solena amplexicaulis* is commonly called creeping cucumber and belongs to the family Cucurbitaceae distributed very seldom in the dry deciduous forest and scrub jungles of Tamil Nadu [9]. The medicinal uses of this species are multifaceted. The local healers of Tamil Nadu and Andhra Pradesh are prescribing this species for many ailments owing to its effective healing property [10]. The traditional healers are prescribing the tubers, leaves, and seeds of this species for various ailments like spermatorrhoea, thermogenics, diuretics, haemorrhoids, and invigorating and it is a very good appetizer and cardiotonic [11]. The whole plant is a potential source of natural antioxidant [12, 13], antidiabetic [10], and antibacterial agent [14] also. As the leaves have good anti-inflammatory activity, it is recommended for inflammation, skin lesions, and skin diseases [15]. Crude leaf juice is used to cure jaundice [16]. Unripe fruits are eaten raw to strengthen the body [17]. The decoction of the root is taken orally to cure stomachache [18]. As the reproductive parts like seeds and tubers are exploited severely for medicinal uses, this species becomes rare sighted in its habitats of Tamil Nadu.

Despite these wide medicinal uses, no information on qualitative account of phytochemicals is available for this species. To address this lacuna, GC-MS studies were undertaken to explore the phytochemical constituents present in the leaf, stem, and tuber parts of* S. amplexicaulis*.

## 2. Materials and Methods

### 2.1. Collection, Identification and Preparation of Plant Materials

The leaf, stem, and tuber parts of* S. amplexicaulis* were collected separately from the thorny scrub jungles of Madukkarai, Coimbatore District, Tamil Nadu, India. The authenticity of the plant was confirmed in Botanical Survey of India, Southern Regional Centre, Coimbatore, by referring to the deposited specimen (Voucher specimen number: CPS 313). They were washed thoroughly in tap water, shade-dried, and then homogenized to fine powder and stored in air tight bottles.

### 2.2. Preparation of Extract

50 g of powdered leaf, stem, and tuber parts of* S. amplexicaulis* was separately extracted with 250 mL methanol at the temperature between 60 and 65°C for 24 h by using soxhlet extractor. The solvent was evaporated by rotary vacuum evaporator to obtain viscous semisolid masses. This semidry methanolic crude extract was subjected to GC-MS analysis.

### 2.3. GC-MS Analysis

GC-MS analysis was carried out on a 5975C Agilent equipped with a DB-5ms Agilent fused silica capillary column (30 × 0.25 mm ID; film thickness: 0.25 *μ*m), operating in electron impact mode at 70 eV. Pure helium (99.999%) was used as carrier gas at a constant flow of 1 mL/min and an injection volume of 1 *μ*L was employed (split ratio is 10 : 1). Mass transfer line and injector temperature were set at 230 and 250°C, respectively. The oven temperature was programmed from 70 (isothermal for 3 min) to 300°C (isothermal for 9 min) at the rate of 10°C/min. Total GC running time was 34 min and the MS detection was completed within 35 min.

By GC-MS, the compounds were separated and then they were eluted from the column and made enter into the detector which was capable of creating an electronic signal. Then they were processed by the computer for generating chromatogram. Then the compound entered into the electron ionization (mass spectroscopy) detector, where they were bombarded with a stream of electrons causing them to break apart into fragments. These fragments were actually charged ions with certain mass. The* m*/*z* (mass/charge) ratio obtained was calibrated from the graph, called the mass spectrum, and is the fingerprint of the molecule.

### 2.4. Identification of the Compounds

To identify the compounds, the extract was assigned for comparison of their retention indices and mass spectra fragmentation patterns with those stored on the computer library and also with the published literature. National Institute of Standards and Technology library sources were also used for matching the identified compounds from the plant materials [19, 20].

## 3. Results

**Figure 1:**
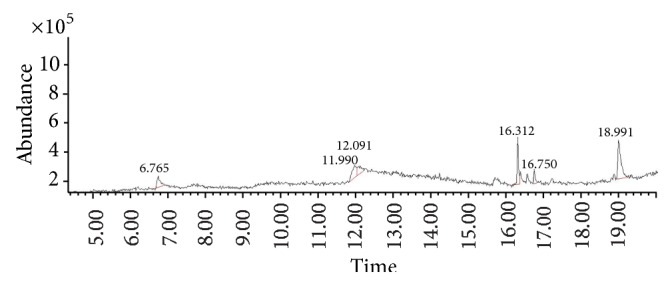
GC-MS chromatogram of methanolic leaf extract of* Solena amplexicaulis*.

**Figure 2:**
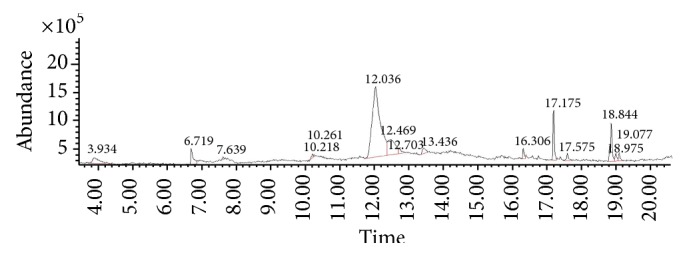
GC-MS chromatogram of methanolic stem extract of* Solena amplexicaulis*.

**Figure 3:**
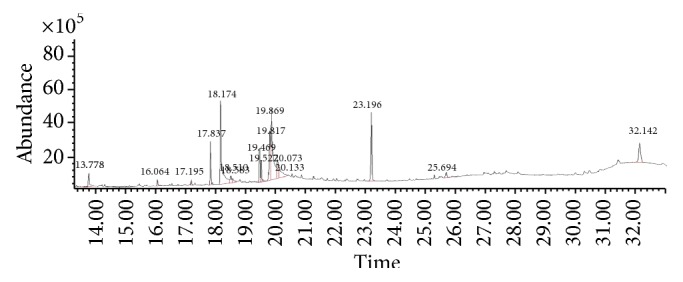
GC-MS chromatogram of methanolic tuber extract of* Solena amplexicaulis*.

**Figure 4:**
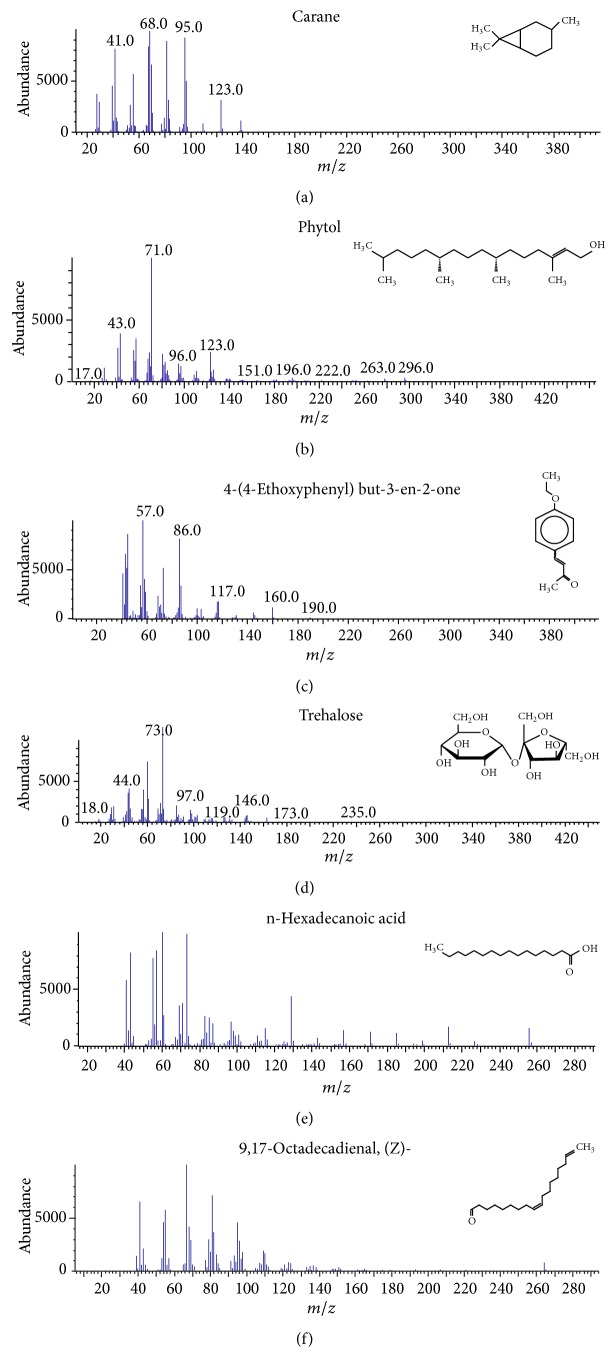
(a) Mass spectrum of carane. (b) Mass spectrum of phytol. (c) Mass spectrum of 4-(4-ethoxyphenyl) but-3-en-2-one. (d) Mass spectrum of trehalose. (e) Mass spectrum of n-hexadecanoic acid. (f) Mass spectrum of 9,17-octadecadienal, (Z)-.

The gas chromatograms of leaf, stem, and tuber parts of* S. amplexicaulis* confirmed the presence of various interesting compounds with different retention times as illustrated in Figures 1, 2, and 3. These compounds were identified through mass spectrometry attached with GC. The identified compounds and their retention time, molecular formula, molecular weight, peak area (%), structure, category of the compound, and activities related with medicinal uses are given in Tables 1, 2, and 3 for leaf, stem, and tuber, respectively. The compound prediction is based on Dr. Duke's Phytochemical and Ethnobotanical Databases. Six compounds were detected in the methanolic leaf extract of* S. amplexicaulis*. Among them, the most prevailing major compounds were phytol, a diterpene (peak area: 38.24%) (Figure 4(a)), carane, a terpene (peak area: 18.76%) (Figure 4(b)), and 1-octanamine, an aliphatic amine (peak area: 16.16%). The methanolic stem extract of* S. amplexicaulis* showed the presence of fifteen different organic compounds. The major phytochemical compounds among them were 4-(4-ethoxyphenyl) but-3-en-2-one, an aliphatic acid (peak area: 56.90%) (Figure 4(c)), trehalose, sucrose (peak area: 11.49%) (Figure 4(d)), hexadecanoic acid, methyl ester, a linoleic acid ester (peak area: 6.52%), and 9-octadecenoic acid (Z)-, methyl ester, another linoleic acid ester (peak area: 6.76%). Fourteen compounds were identified in the methanolic tuber extract. In this account, 9,17-octadecadienal (Z)-, an unsaturated aldehyde (peak area: 21.77%) (Figure 4(e)), n-hexadecanoic acid, a palmitic acid (peak area: 21.75%) (Figure 4(f)), phthalic acid, di(2-propylpentyl) ester, a dicarboxylic acid ester (peak area: 9.48%), and 9,12-octadecadienoic acid (Z,Z)-, a linolenic acid (peak area: 9.35%) were the major phytochemicals on the basis of quantity.

## 4. Discussion

The gas chromatogram shows that the relative concentrations of various compounds are getting eluted as a function of retention time. The height of the peaks indicates the relative concentrations of the compounds present in the plant. The mass spectrometer analyzes of the compounds eluted at different times to identify the nature and structure of the compounds. The large compound fragments into small compounds give rise to appearance of peaks at different *m*/*z* ratios. These mass spectra are fingerprint of that compound which can be identified from the data library.

Generally, the reliability of medicinal plant for its usage is evaluated by correlating the phytochemical compounds with their biological activities [21]. In the present study, the GC-MS analysis of the methanolic extracts of leaf, stem, and tuber parts of* S. amplexicaulis* altogether showed the presence of 35 compounds. In this account, the leaf extract contained six compounds among them, phytol (38.24%) is having anticancer, antioxidant, anti-inflammatory, antitumor, antimicrobial, diuretic, and chemopreventive properties and used in vaccine formulations [22, 23]. The other compound, carane (18.76%) is having antifeedant and antioxidant properties [24, 25]. The methanolic stem and tuber extracts showed the presence of greater number of 14 and 15 compounds, respectively. The six phytoconstituents, namely, undecane, taurolidine, trehalose, hexadecanoic acid methyl ester, 9-octadecenoic acid (Z)-, methyl ester, and benzaldehyde, 2-nitro-, diaminomet hylidenhydrazone in stem extracts have possessed medicinal properties [26]. Undecane, an alkane, is an antimicrobial agent, used as carcinogen [27, 28]. Similarly, the other compound, taurolidine, a taurine amino acid derivative, has antimicrobial, antilipopolysaccharidal, and antitumor properties [29, 30]. The sucrose compound, trehalose, is used for the treatment of amyloidosis [31]. The linoleic acid esters present in the stem, hexadecanoic acid methyl ester, are reported to have anti-inflammatory, cancer preventive, hepatoprotective, antiarthritic, and anticoronary properties. The other linoleic acid ester, 9-octadecenoic acid (Z)-, methyl ester, is also having anti-inflammatory, antiandrogenic, and anemiagenic properties [32]. The nitrogen compound, benzaldehyde, 2-nitro-, diaminomet hylidenhydrazone, is known to have the property of curing infectious diseases by its antimicrobial activity. In the tuber extracts, the compounds identified, namely, 10,13-octadecadienoic acid methyl ester, trans-13-octadecenoic acid, methyl ester, and 9,12-octadecadienoic acid (Z,Z)-, are possessed with anti-inflammatory and cancer preventive characters. The two compounds, namely, tetradecanoic acid and n-hexadecanoic acid, are antioxidants. The phthalic acid, 1,2-benzenedicarboxylic acid, bis(2-methylpropyl) ester, is used in the preparation of perfumes and cosmetics. The unsaturated alcoholic compound, 9,17-octadecadienal, (Z)-, is reported to have antimicrobial property [33]. The study species* S. amplexicaulis* is endowed with various medicinal properties maybe due to the presence of all these compounds described. In a similar fashion, certain traditional medicinal plant species of Cucurbitaceae have been analyzed phytochemically by using GC-MS and suggested for drug preparation after succeeding in clinical trials [34, 35]. The therapeutic properties of the other compounds in all the three parts of* S. amplexicaulis* were not yet reported.

Our investigation through the present study revealed that the species* S. amplexicaulis* is a reliable source of bioactive compounds like fatty acid esters, alcohols, hydrocarbons, alkanes, amines, terpenes, and sugars that justify the traditional usage of this species [16–18] by the local healers in Coimbatore and Tirupur districts of Tamil Nadu, India, for various ailments. As GC-MS is the first step towards understanding the nature of active principles [36, 37], further investigation in this species is suggested for the development of novel drugs.

## Figures and Tables

**Table 1:** Compounds identified in the methanolic leaf extract of *Solena amplexicaulis* by GC-MS.

S. number	Name of the compound	RT	Molecular formula	Molecular weight	Peak area %	Structure	Category of the compound	Activity∗
1	Hexahydropyridine, 1-methyl-4-[4,5-dihydroxyphenyl]-	6.761	C_12_H_17_NO_2_	207.12	10.75	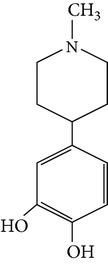	Aromatic piperidine	No activity reported

2	1-Octanamine	11.990	C_8_H_19_N	129.24	16.16	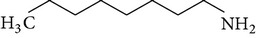	Aliphatic amine	No activity reported

3	1-Tetradecanamine	12.091	C_14_H_31_N	213.40	10.24	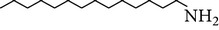	Aliphatic amine	No activity reported

4	Carane	16.317	C_10_H_18_	138.24	18.76	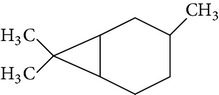	Terpene	Antifeedant, antioxidant

5	Pentane-2,4-dione, 3-(1-adamantyl)	16.753	C_15_H_22_O_2_	234.33	5.85	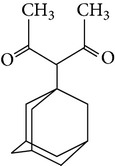	Aliphatic diketone	No activity reported

6	Phytol	18.990	C_20_H_40_O	296.53	38.24	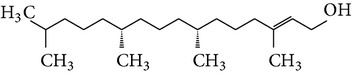	Diterpene	Anticancer, antioxidant, anti-inflammatory, diuretic, antitumor, chemopreventive, antimicrobial, use in vaccine formulations

∗Source: Dr. Duke's Phytochemical and Ethnobotanical Databases (online database).

**Table 2:** Compounds identified in the methanolic stem extract of *Solena amplexicaulis* by GC-MS.

S. number	Name of the compound	RT	Molecular formula	Molecular weight	Peak area %	Structure	Category of the compound	Activity∗
1	1,3-Cyclopentanedione	3.929	C_5_H_6_O_2_	98.09	4.47	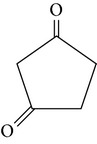	Cyclic diketone	No activity reported

2	Undecane	6.718	C_11_H_24_	156.30	3.92	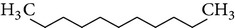	Alkane	Antimicrobial agents, transducer for immunosensor and its method of production. carcinogens, enzyme inhibitors, solvents

3	1,2,4-Triazino [5,6-E] [1,2,4]-triazine-3,6-dione, hexahydro-	7.633	C_4_H_8_N_6_O_2_	172.14	0.36	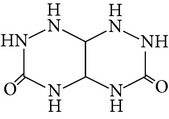	Triazine	No activity reported

4	4-Hydroxyphenyl 3-nitrobenzoate	10.218	C_13_H_9_NO_5_	259.21	0.52	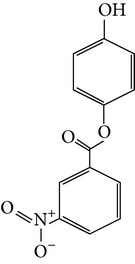	Aromatic nitro compound	No activity reported

5	Taurolidine	10.261	C_7_H_16_N_4_O_4_S_2_	284.35	0.17	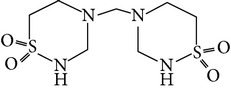	Taurine amino acid derivative	Antimicrobial, anti-lipopolysaccharide, anti-tumor properties, anti-infective agents, antineoplastic agents

6	4-(4-Ethoxyphenyl) but-3-en-2-one	12.033	C_12_H_14_O_2_	190.24	56.90	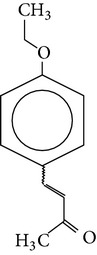	Aliphatic acid	No activity reported

7	Trehalose	12.469	C_12_H_22_O_11_	342.29	11.49	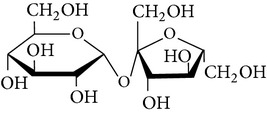	Sucrose	Treat amyloidosis (prevent the deposition of amyloid protein in the body)

8	d-Glycero-d-tallo-heptose	12.701	C_7_H_14_O_7_	210.18	1.68	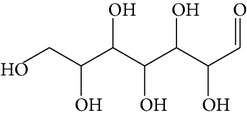	Aldo heptose	No activity reported

9	Benzaldehyde, 6-hydroxy-4-methoxy-2,3-dimethyl-	13.442	C_10_H_12_O_3_	180.20	1.71	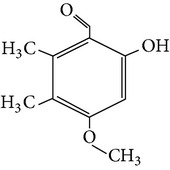	Aromatic benzaldehyde	No activity reported

10	9-Tetradecen-1-ol, acetate, (Z)-	16.303	C_16_H_30_O_2_	254.40	1.40	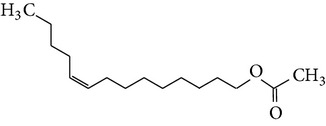	Aliphatic ester	No activity reported

11	Hexadecanoic acid, methyl ester	17.174	C_17_H_34_O_2_	270.45	6.52	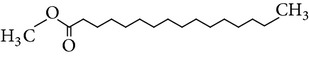	Linoleic acid ester	Anti-inflammatory, hypocholesterolemic, cancer preventive, hepatoprotective, nematicide, insectifuge, antihistaminic, antieczemic, antiacne, alpha reductase inhibitor, antiandrogenic, antiarthritic, anticoronary

12	1-Methyl-3-ethyladamantane	17.581	C_13_H_22_	178.31	1.37	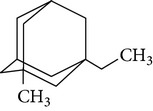	Bicyclic alkane	No activity reported

13	9-Octadecenoic acid (Z)-, methyl ester	18.844	C_19_H_36_O_2_	296.48	6.76	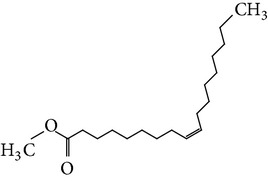	Linoleic acid ester	Anti-inflammatory, antiandrogenic cancer preventive, dermatitigenic hypocholesterolemic, 5-alpha reductase inhibitor, anemiagenic, insectifuge

14	Benzaldehyde, 2-nitro-, diaminomet hylidenhydrazone	18.975	C_8_H_9_N_5_O_2_	207.18	1.42	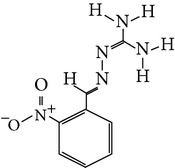	Nitrogen compound	Antimicrobial

15	Heptadecanoic acid, 10-methyl-, methyl ester	19.077	C_19_H_38_O_2_	298.50	1.29	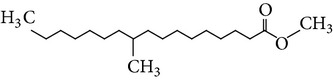	Fatty ester	No activity reported

∗Source: Dr. Duke's Phytochemical and Ethnobotanical Databases (online database).

**Table 3:** Compounds identified in the methanolic tuber extract of *Solena amplexicaulis* by GC-MS.

S. number	Name of the compound	RT	Molecular formula	Molecular weight	Peak area %	Structure	Category of the compound	Activity∗
1	Dodecanoic acid	13.776	C_12_H_24_O_2_	200.31	2.40	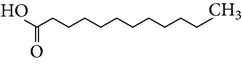	Fatty acids	No activity reported

2	Tetradecanoic acid	16.071	C_14_H_28_O_2_	228.37	0.95	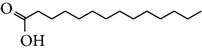	Myristic acid	Antioxidant, cancer preventive, nematicide, hypocholesterolemic, lubricant

3	1,2-Benzenedicarboxylic acid, bis(2-methylpropyl) ester	17.189	C_16_H_22_O_4_	278.34	0.74	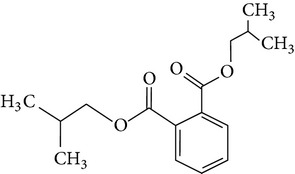	Phthalic ester	Used in preparation of perfumes and cosmetics, plasticized vinyl seats on furniture, cars, and clothing including jackets, raincoats, and boots and used in textiles, as dyestuffs, cosmetics, and glass making

4	Pentadecanoic acid, 14-methyl-, methyl ester	17.842	C_17_H_34_O_2_	270.45	4.61	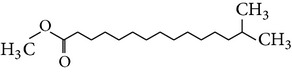	Fatty ester	No activity reported

5	n-Hexadecanoic acid	18.176	C_16_H_32_O_2_	256.42	21.75	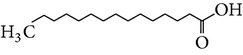	Palmitic acid	Antioxidant, hypocholesterolemic, nematicide, pesticide, lubricant, hemolytic inhibitor, antiandrogenic

6	Cystodytin	18.510	C_22_H_19_O_3_N_3_	373.78	1.58	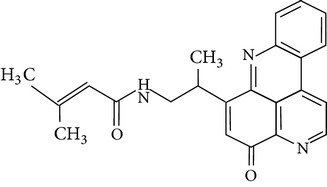	Aromatic alkaloid	Antiproliferative activity in human tumor cell lines

7	1-Decanol, 2-hexyl-	18.583	C_16_H_34_O	242.44	1.21	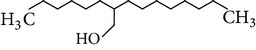	Aliphatic alcohols	Antimicrobial

8	10,13-Octadecadienoic acid, methyl ester	19.469	C_19_H_34_O_2_	294.47	4.72	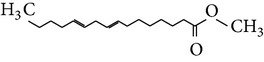	Linoleic acid esters	Anti-inflammatory, hypocholesterolemic, cancer preventive, hepatoprotective, nematicide, insectifuge, antieczemic, anticancer, antiarthritic, insectifuge, antihistaminic, anticoronary

9	*trans*-13-Octadecenoic acid, methyl ester	19.527	C_19_H_36_O_2_	296.48	3.55	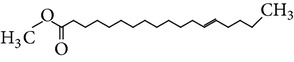	Linoleic acid esters	Anti-inflammatory, antiandrogenic, cancer preventive, dermatitigenic, irritant, antileukotriene—D4, hypocholesterolemic, 5-alpha reductase inhibitor, anemiagenic, insectifuge, flavor

10	9,12-Octadecadienoic acid (Z,Z)-	19.817	C_18_H_32_O_2_	280.44	9.35	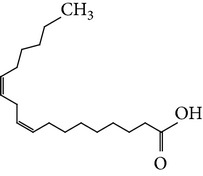	Linolenic acid	Anti-inflammatory, hypocholesterolemic, cancer preventive, insectifuge, antiarthritic, hepatoprotective, antiandrogenic, nematicide, antihistaminic, antieczemic

11	9,17-Octadecadienal, (Z)-	19.876	C_18_H_32_O	264.44	21.77	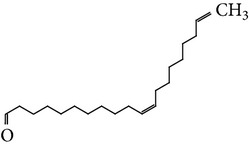	Unsaturated aldehyde	Antimicrobial

12	Phthalic acid, di(2-propylpentyl) ester	23.201	C_24_H_38_O_4_	390.55	9.48	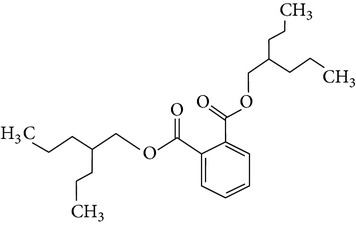	Dicarboxylic acid ester	Oral toxicity during pregnancy and sucking in the Long-Evans Rat

13	Anthracene, 9-ethyl-9,10-dihydro-10-t-butyl-	25.699	C_20_H_24_	264.40	1.26	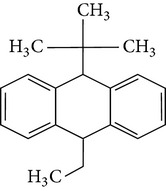	Hydrocarbons	No activity reported

14	4-Dehydroxy-N-(4,5-methylenedioxy-2-nitrobenzylidene) tyramine	32.148	C_16_H_14_N_2_O_4_	298.29	6.72	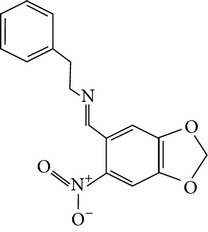	Tyramine derivative	No activity reported

∗Source: Dr. Duke's Phytochemical and Ethnobotanical Databases (online database).
